# Delayed Presentation of Gastric Volvulus with Gastric Outlet Obstruction after Laparoscopic Sleeve Gastrectomy

**DOI:** 10.1155/2022/2157522

**Published:** 2022-04-08

**Authors:** Esam Batayyah, Waed Yaseen, Shatha Althobaiti

**Affiliations:** Al Noor Specialist Hospital, Makkah, Saudi Arabia

## Abstract

**Background:**

Globally among bariatric procedures, SG popularity has grown significantly. New complications arise as a result of the rapid growth in the numbers of LSG. Once SG has been performed, the stomach is left with no fixations along the entire greater curvature, which may predispose to volvulus. *Case Report*. The 44-year-old patient had laparoscopic sleeve gastrectomy 2 years and 6 months before presentation in our emergent department complaining of episodic intolerance to oral intake for 3 months before presentation. Gastrografin swallow study showed gastric outlet obstruction. The patient was taken to the operation room for laparoscopic exploration, gastric untwisting.

**Conclusion:**

This case highlights a rare complication of SG which has been inconsistently addressed in the literature. Awareness of such complications would help surgeons to widen their differential diagnosis of postoperative sleeve gastrectomy early complications to manage them effectively. The video of the procedure showing the intraoperative finding can be accessed at.

## 1. Introduction

Since its introduction by Gagner in 1999, laparoscopic sleeve gastrectomy (LSG) has rapidly emerged as a single-stage operation for the treatment of morbid obesity [1]. Morbid obesity can be managed effectively by LSG as a standalone procedure. LSG functions as a restrictive procedure besides removing the ghrelin-producing portion of the stomach, thus it may cause early satiety [2].

In the modern series, the overall mortality rate of LSG is approximately 0 to 1.2 percent; the overall morbidity rates range from 0 to 17.5 percent [3]. The most common complications of SG are leaks, abscesses, hemorrhages, and strictures where each of them represents (0.7%) [2].

Globally among bariatric procedures, SG popularity has grown significantly and now encompasses 27.8% of surgeries performed in the past 8 years [4]. New complications arise as a result of the rapid growth in the numbers of LSG. Once SG has been performed, the stomach is left with no fixations along the entire greater curvature, which may increase the possibility of the volvulus [5, 6].

Awareness of such complications would aid in wisely selecting the candidates for each bariatric procedure and immediately diagnose and effectively manage those postoperative complications.

## 2. Case Presentation

**Figure 1:**
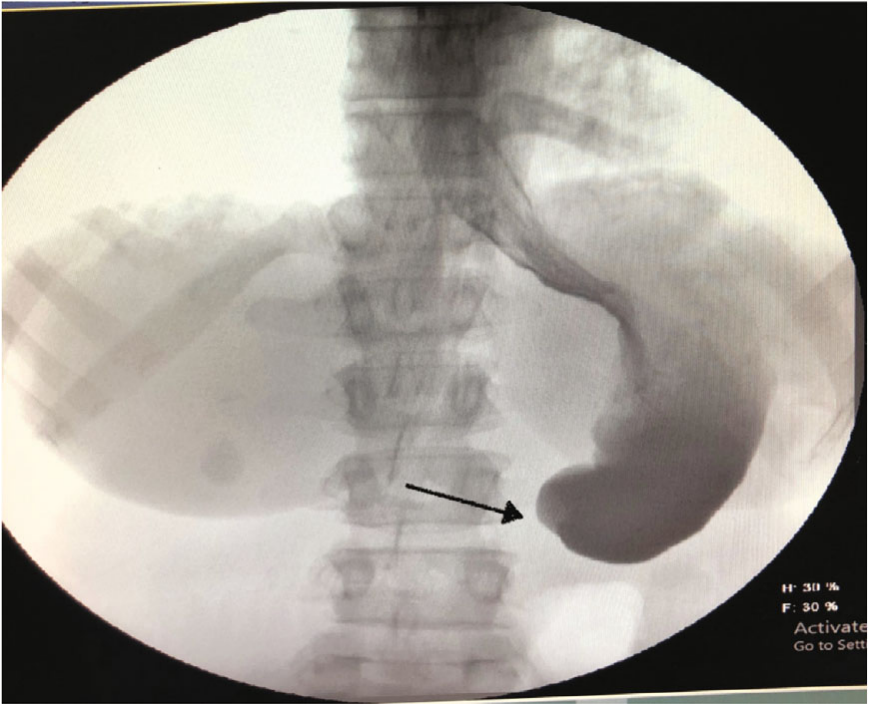
Gastrografin swallow study showing gastric outlet obstruction with mild gastroesophageal reflux as seen on fluoroscopy.

The patient is a 44-year-old female post sleeve gastrectomy 2 years and 6 months before presentation to our emergent department with a history of episodic intolerance to oral intake for 3 months, no abdominal distention or change in bowel habit. On examination, abdomen has no distention, scars of previous surgery, soft and lax, no tenderness. Laboratory investigations were remarkable for electrolyte disturbance. Gastrografin swallow study (Figure 1) showed gastric outlet obstruction with mild gastroesophageal reflux as seen on fluoroscopy.

**Figure 2:**
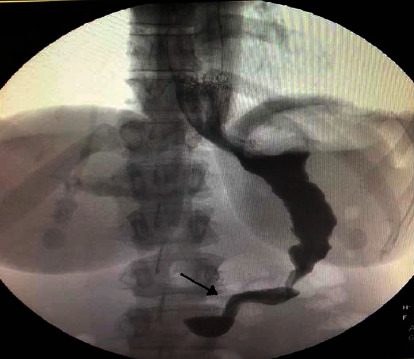
Postoperative gastrografin swallow study showed contrast is passing through the duodenum into small loops. No gastroesophageal reflux was seen on fluoroscopy.

The patient was taken to the operation room for laparoscopic exploration and gastric untwisting (https://www.youtube.com/watch?app=desktop&v=9D68sYBsALQ&feature=youtu.be), with the finding of adhesion of the omentum and stomach to the liver, twisting of the previously sleeved stomach, two fibrous bands just below the fundus, and at the incisura, distention of the fundus and the antrum of the stomach. Adhesions were dissected, and the stomach was released from the liver. The fibrous bands were released. EGD was introduced and passed to the antrum. Histopathology was taken from the band, which came as an unremarkable omental tissue. Follow-up gastrografin (Figure 2) showed contrast is passing through the duodenum into small bowel loops.

No gastroesophageal reflux was seen on fluoroscopy. The patient improved postsurgery and tolerating orally.

## 3. Discussion

Gastric volvulus is defined as a rotation of the stomach around the anatomic axes and is a rare condition. Organo-axial and mesenteric-axial are the two forms of gastric volvulus [7]. Axis in organo-axial is longitudinal and passes through the pylorus and gastroesophageal junction where axis in mesenteric-axial is transverse and passes through the middle of the stomach. The stomach is fixed to its normal poison in the abdomen by the ligaments between the stomach and its adjacent oranges namely gastrosplenic, gastrophrinic, gastrohepatic, and gastrocolic ligaments [7]. The stomach might be prone for volvulus as long as there is laxity in the gastric fixation or incorrect positioning of the stomach postsurgical manipulation [7].

Usually, after placement of the band in laparoscopic gastric banding, several gastric volvulus cases have been reported [4, 6]. They recommended during band replacement to not dissect the posterior wall of the stomach. Del Castillo et al. reported a gastric volvulus case after laparoscopic sleeve gastrectomy [8]. As in our case, we found the twisting of the stomach on its oregano-axial axis.

## 4. Conclusions

In the literature, technical complications in relation to LSG seem to be underreported. Awareness of such complications would aid in wisely selecting the candidates for each bariatric procedure and be prepared to immediately diagnose and effectively manage those postoperative complications. Bariatric surgeons should take into consideration this diagnosis and explore its symptoms in LSG patients at postoperative follow-up visits.
